# Robust Resilience and Substantial Interest: A Survey of Pharmacological Cognitive Enhancement among University Students in the UK and Ireland

**DOI:** 10.1371/journal.pone.0105969

**Published:** 2014-10-30

**Authors:** Ilina Singh, Imre Bard, Jonathan Jackson

**Affiliations:** 1 Department of Social Science, Health & Medicine, Kings College London, London, United Kingdom; 2 Department of Methodology, London School of Economics & Political Science, London, United Kingdom; University of Chicago, United States of America

## Abstract

Use of ‘smart drugs’ among UK students is described in frequent media reports as a rapidly increasing phenomenon. This article reports findings from the first large-scale survey of pharmacological cognitive enhancement (PCE) among students in the UK and Ireland. Conducted from February to September 2012, a survey of a convenience sample of 877 students measured PCE prevalence, attitudes, sources, purposes and ethics. Descriptive and logistic regression statistical methods were used to analyse the data. Lifetime prevalence of PCE using modafinil, methylphenidate or Adderall was under 10%, while past regular and current PCE users of these substances made up between 0.3%–4% of the survey population. A substantial majority of students was unaware of and/or uninterested in PCE; however about one third of students were interested in PCE. PCE users were more likely to be male, British and older students; predictors of PCE use included awareness of other students using PCEs, ADHD symptomatology, ethical concerns, and alcohol and cannabis use. The survey addresses the need for better evidence about PCE prevalence and practices among university students in the UK. We recommend PCE-related strategies for universities based on the survey findings.

## Introduction

### Prevalence of pharmacological cognitive enhancement

The past five years have seen increasing debate about cognitive enhancement, emerging initially in the United States and then more recently in the United Kingdom and Europe. A general definition of cognitive enhancement is provided in Hildt & Franke [1]:

Cognitive enhancement is the use of drugs, biotechnological strategies or other means by healthy individuals aiming at the improvement of cognitive functions such as vigilance, concentration or memory without any medical need. [1:2].

The use of prescription drugs for non-medical purposes has driven some of the controversy over cognitive enhancement. The academic literature on pharmacological cognitive enhancement (PCE) has focused primarily on three pharmacological substances: methylphenidate (e.g. Ritalin) and its related compounds; mixed amphetamine salts, traded in the United States under the name Adderall; and modafinil (Provigil). Methylphenidate and mixed amphetamine salts are psychostimulants, and are the most common forms of pharmacological treatment for Attention Deficit/Hyperactivity Disorder (ADHD). Modafinil is used as a treatment for narcolepsy.

PCE has invoked increasing media scrutiny in many countries around the world [2]–[4], with special emphasis placed on the non-medical use of prescription drugs by university students. Several national ethics advisory bodies have addressed PCE (5), including the US President's Council on Bioethics, the UK Parliament, and the Italian National Bioethics Commission [6]–[8].

A salient topic in the debate is non-medical use of prescription drugs as ‘smart drugs’ or ‘study aids’ among university students. PCE prevalence data is available primarily from research among US university students, in which estimates of non-prescription stimulant drug use range from 5%–35% [9]–[10], with members of fraternities and sororities often showing the highest rates [11].

Documentation of PCE prevalence in Europe has been both less systematic and less comprehensive than in the US. Looking across a range of different studies, PCE prevalence estimates in different EU Member States range from 0.8% to 16%, depending on country, university and type of drug used [12]–[19]. Moreover, variation in study sample and methods, and research design quality, complicates comparative understanding of PCE prevalence across the EU [20].

Research on PCE prevalence in the UK is particularly sparse. The only published study to date is an investigation of prescription drug abuse that includes PCE as one form of drug misuse. From a sample of 1614 students and 489 university staff at a university in Wales [19], a total of 37 students (0.02%) reported having used stimulant drugs without a prescription; of these respondents, three reported having used prescription stimulants in order to study.

The other source of systematic evidence available on PCE prevalence in the UK derives from a population survey conducted by the Wellcome Trust (http://www.wellcome.ac.uk/About-us/Publications/Reports/Public-engagement/WTX058859.htm). The Wellcome Monitor, which included seven questions on cognitive enhancement, found low PCE prevalence in the general population. Approximately 2% of adult respondents and 1% of young people aged 14–18 admitted to PCE using Ritalin or Adderall. No respondents had used modafinil for PCE. One third of both adults and young people held that regular or occasional use of pharmacological cognition enhancement was acceptable.

In the absence of research evidence, journalism is the most widely cited source of information about UK PCE prevalence. In 2009 the student newspaper at Cambridge University, *The Varsity*, surveyed 1000 students and found a 10% PCE prevalence rate. The survey queried students about “taking medication without prescription to help them work” [21]. Information about the survey is limited to a short article in the student paper.

### Limitations of PCE survey evidence

While large-scale surveys of PCE can in principle provide us with a high level understanding of PCE prevalence, published PCE surveys tend to have several limitations. Prevalence data are often misrepresented because drug use practices and purposes are not elicited as part of the survey. Drug use practices are important because lifetime prevalence can easily be misrepresented as frequent and on-going use when an individual has in fact used the drug only once or twice. In turn, this can lead to overestimates of current PCE prevalence, and can help to foster a false picture of PCE as a widespread and ongoing practice [20]. Drug use purposes are also important to distinguish in a PCE survey. As Partridge [22] has argued, reported misuse of prescription stimulants is often assumed to mean that misuse is aimed specifically at cognition enhancement, when the true purpose may instead be recreational.

Surveys have rarely investigated the ethical dimensions of PCE. Evidence from qualitative studies in the US suggests that PCE users tend to conceive of PCE as being both safe and morally unproblematic [23]–[25]. A small interview study among German university students found that ethical considerations were a relatively low priority in PCE decision-making. Medical and legal dimensions played a more important role than ethical considerations in students' reasoning about the use of caffeine as a ‘smart drug’ as compared to PCE [26]. Two studies with Cambridge University students found that participants condemned PCE when there were long-term negative effects on health and when PCE was seen to confer an unfair advantage in exam situations [27].

### Rationale for the current study

Despite the lack of evidence for PCE prevalence in the UK, there is an increasing level of concern and activity around PCE, encouraged in the media and extending to public debate and Parliamentary and Home Office activity [28]. Within these debates, prevalence estimates for PCE in the UK are derived from poor quality sources, as outlined above. Characteristics of UK PCE users and practices, as well as PCE sources, are the subject of widespread speculation [3]. Attitudes toward PCE in the UK also lack systematic investigation.

The aim of the current study was to conduct a national survey of PCE among UK and Irish university students in order to gain better understanding of PCE in a population hypothesized to be active PCE users.

## Methods

A survey of a convenience sample of students enrolled in universities in the UK and Ireland was conducted from February to September 2012. Participants were recruited using on-line methods, including social media sites and national university student mailing lists.

The survey was developed on the basis of a review of the available empirical studies of PCE, as well as a series of focus groups involving 70 university students. Before launching, the survey was piloted with 13 university students and questions were revised on the basis of their feedback. The final survey included measures of knowledge of smart drugs, drug use patterns (prevalence, last-year and last-month prevalence, drug use motivations and frequency), eventual prescription use of cognition enhancers (which also included questions about non-prescribed ways of use as well as diversion), questions on ethics, and two psychological instruments: the 6-item World Health Organisation (WHO) version of the Adult ADHD Self-report Scale (http://www.hcp.med.harvard.edu/ncs/asrs.php) and the Rosenberg Self-Esteem Scale (https://personality-testing.info/tests/RSE.php).

The survey was conducted using Surveygizmo (www.surveygizmo.com) under a domain (www.thesmartdrugstudy.com) purchased and set up for the purposes of the project.

Respondents were presented with the following definition:

Smart drugs, also known as cognitive enhancers, are prescription and non-prescription substances that people use to improve their cognitive functioning and performance.

From a list containing 27 substances (in randomized order) respondents were asked to mark those they considered to be ‘smart drugs’ based on the above definition; they also had the option to enter additional drugs into a textbox if they considered an important substance to be missing from the list. For the purposes of this study we considered the following drugs to be cognition enhancers: methylphenidate, modafinil, Adderall, donepezil, piracetam, and atomoxetine.

When assessing drug use prevalence we distinguished among 6 different categories of use for each of the 27 drugs listed on the survey:

1. I have never heard of it
2. I never tried it and was never interested
3. I never tried it but considered doing so
4. I tried it a few times in the past
5. I used it regularly in the past
6. I use it nowadays

To differentiate substance use groups, we created four distinct and mutually exclusive categories of user groups for the most commonly identified PCEs (modafinil, methylphenidate and Adderall).

1. Respondents who indicated that they had never heard of, or that they were not interested in methylphenidate, modafinil and Adderall
2. Respondents who indicated their interest in at least one of three substances but had never tried any of the them
3. Respondents who had used methylphenidate, modafinil, or Adderall but not for the purpose of enhancing cognitive performance (non-PCE-type use)
4. Respondents who had used at least one smart drug for the purpose of enhancing cognitive performance (PCE users).

To obtain a detailed understanding of drug use purposes, respondents were asked to indicate all the relevant motivations for using each substance, by choosing from a list containing 18 options or entering their individual answer into an open-ended textbox.

We asked those respondents who indicated an interest in modafinil, methylphenidate or Adderall, but who had not actually used these substances, to provide reasons why they had not used the drugs in question. Respondents described their reasons in a text-entry box on the survey. Answers were coded by two independent coders into 14 previously agreed categories (see www.thesmartdrugstudy.com for further information). When respondents gave several reasons why interest had not converted to use, each reason was individually coded and categorised. When responses were coded into different categories by the two coders, then the final result is the rounded-up average of the two coders' results.

Respondents were asked whether PCE use by students in an academic setting is ethically problematic. Three more specific questions were also asked: whether PCE use by students is like doping in sports, whether PCE use by students constitutes cheating, and if students feel pressure to use PCEs at university. A Likert-type scale was used to query respondents on ethical issues. Responses on each question were analysed for the four PCE user-types described earlier.

Statistical analysis was conducted using Stata 11, generating standard cross-tabulations, frequency tables and mean comparisons.

We also fitted a series of multinomial logistic regression models to examine the predictors of drug usage for modafinil, methylphenidate, Adderall and caffeine, in which we differentiated among the four user groups identified above. We used ordinal logistic (or proportional odds) modelling to estimate the factors associated with moving up or down the four user group categories for each drug. The potential predictors were: gender, age (17–20, 21–24, 25–29 and 30+), ethnicity (White British, other White, and non-White), alcohol use, cannabis use, awareness of other people at university using smart drugs, ADHD score, self-esteem score, and beliefs about the ethics of using smart drugs to improve academic performance.

The statistical technique groups adjacent values of the ordinal response into two groups, “high" (i.e. higher-numbered) and “low", divided at each level j = 1;:::;C-1 in turn. For drug use and awareness, there are three such groupings:

- used [high] vs. (considered, uninterested, unaware) [low]
- (used, considered) [high] vs. (uninterested, unaware) [low]
- (used, considered, uninterested) [high] vs. (unaware) [low]

The regression coefficient of an explanatory variable X is then interpreted as the log odds ratio (and its exponential as the odds ratio) associated with a one-unit increase in X, for the choice of the high rather than low value in these dichotomies, with the same odds ratio applied to each way of grouping the response levels. Blocks of variables were added one at a time resulting in six models (Model I includes demographic variables, Model II adds alcohol use and cannabis use, Model III adds awareness of other people at university using smart drugs, and so on) to assess the effect of incrementally adjusting for other factors.

The proportional odds assumption was tested using the Brant test [29]. The test indicated no problems for all variables but the ‘beliefs about the ethics of using smart drugs to improve academic performance’ variable, added in Model VI for each of the drugs. Because the other predictors in the fitted ordered logistic models conformed to the proportional odds assumption, and because the findings from the multinomial logistic regression models indicated the same pattern of relationships, (controlling for ethical beliefs), we proceed with discussion of results from this analysis. We also interpret the results from the fitted multinomial logistic regressions when it comes to beliefs about the ethics of using smart drugs to improve academic performance in light of the Brant test.

## Results

The final sample size was 877. Two respondents who completed the survey were excluded because they reported taking a fake drug called ‘Relevin’ [30]. We also dropped 14 respondents from the sample who said they had used cognitive enhancers for a medical indication and had a legitimate prescription.

Table 1 outlines the demographic profile of the sample. 75% of students in the sample reported being undergraduates. Participation was almost equally split between males and females. The mean age of participants was 22.7 years old. Participants were drawn from 104 universities in the UK and Ireland; a majority of respondents (79%) reported enrolment at a Russell Group university. The Russell Group is an association of the leading research universities in the United Kingdom. Our sample includes students from 23 of the 24 Russell Group member institutions. Universities most frequently represented in the survey were: Bristol University (*n* = 161), Manchester University (*n* = 96), Cardiff University (*n* = 88), London School of Economics (*n* = 86), Cambridge University (*n* = 77), Oxford University (*n* = 34) and University College London (*n* = 32).

**Table 1:** Participant characteristics (*n* = 877).

	%	*n*
Gender		
Male	46%	403
Female	53%	467
Not recorded	1%	7
Degree		
Undergraduate	75%	660
Postgraduate	22%	192
Other	3%	25

### Smart drugs

The five substances most frequently identified as ‘smart drugs’ were caffeine pills, methylphenidate, energy drinks, vitamin supplements, and modafinil (See Table 2 below for complete list). A small proportion of the sample (2%) was able to identify all 6 substances our study considered to be cognition enhancers, while 44% of respondents identified none as a smart drug.

**Table 2:** What is a smart drug? (*n* = 877).

Substance	% of respondents identifying it as a smart drug	Substance	% of respondents identifying it as a smart drug
1. caffeine pills	42.1%	15. LSD	6.2%
2. methylphenidate	41.5%	16. MDMA	5.7%
3. energy drinks	33.9%	17. DMT	4.3%
4. vitamin supplements	29.2%	18. magic mushrooms	4.3%
5. modafinil	25.9%	19. sleeping pills	4.2%
6. Adderall	25.3%	20. Relevin	3.9%
7. speed	22.4%	21. crystal meth	3.8%
8. piracetam	13.1%	22. mephedrone	3.3%
9. ephedrine	9.2%	23. alcohol	2.6%
10. marijuana	9.3%	24. pain killers	2.6%
11. donepezil	8.9%	25. ketamine	2.5%
12. tobacco	8.3%	26. tranquilizers	22.2%
13. cocaine	7.8%	27. heroin	1.5%
14. atomoxetine	6.8%		

### PCE prevalence

We measured lifetime prevalence for the three prescription drugs most commonly used for PCE (methylphenidate, modafinil and Adderall). We also conducted a comparative analysis with caffeine, which a majority of students identified as a cognitive enhancer. Table 3 presents the breakdown for these four substances.

**Table 3:** **S**mart Drug Prevalence (n = 877).*

		methylphenidate *n* (%)	modafinil *n* (%)	Adderall *n* (%)	caffeine pills *n* (%)
**Unaware**		154 (17.6%)	514 (58.6%)	434 (49.5%)	13 (1.5%)
**Uninterested**		507 (57.8%)	213 (24.3%)	304 (34.7%)	302 (34.4%)
**Considered**		164 (18.7%)	80 (9.1%)	110 (12.5%)	129 (14.7%)
**Past occasional**	**PCE use**	22 (2.5%)	18 (2.1%)	12 (1.4%)	109 (12.4%)
	**Other use**	15 (0.7%)	10 (1.1%)	7 (0.8%)	141 (16.1%)
**Past regular**	**PCE use**	6 (1%)	6 (0.7%)	3 (0.3%)	49 (5.6%)
	**Other use**	2 (0.2%)	2 (0.2%)	2 (0.2%)	43 (4.9%)
**Current**	**PCE use**	7 (0.8%)	30 (3.4%)	3 (0.3%)	55 (6.3%)
	**Other use**	0 (0%)	4 (0.5%)	2 (0.2%)	36 (4.1%)

*Table 3 contains synthesized information from two questions. Prevalence – based on the six options listed – and purposes, focusing on PCE. Respondents could choose from 17 other purposes besides PCE; a subset of the data on other purposes is reported in Table 4. ‘PCE use’ was defined as a respondent who indicated PCE alone or in any combination; ‘other use’ was defined as a respondent who did not indicate PCE, but did indicate any other purpose.

Lifetime prevalence for methylphenidate was 5.9%. The drug was unknown to almost 18% of our sample; 19% had considered trying it and 58% reported that they had never had interest in trying it. About 4% of users had taken methylphenidate for PCE, while about 1% had taken it for non-PCE use.

Modafinil was simultaneously the most unknown and the most frequently used cognition enhancer. Lifetime prevalence for modafinil was 8%. Lifetime prevalence for PCE use was 6.2%, which suggests that modafinil was used primarily for PCE purposes by this sample of students. Almost 59% of students had never heard of modafinil, while about 24% said they were never interested in trying it. Nine percent of students had considered trying modafinil.

Lifetime prevalence for Adderall was 3.2% overall and 2% for PCE use. Around 50% of respondents were unaware of Adderall; over a third were uninterested in it; and about 13% had considered using the drug.

In the case of the psychostimulants (methylphenidate and Adderall) there was a marked difference between the number of respondents who had tried the drug in the past and those who were current users. Respondents were five times more likely to be past users of methylphenidate than current users. Similarly, respondents were four times more likely to be past users of Adderall than current users. Modafinil use patterns differed from methylphenidate and Adderall use patterns; there was a slightly higher number of current users than past users of modafinil.

In comparison with the prescription medications, the lifetime prevalence for caffeine pills was 49.4%, with 24.3% lifetime prevalence for cognitive enhancement. A very small proportion of respondents were unfamiliar with caffeine pills (1.5%), while over 10% identified as current users, with current users choosing caffeine pills for cognitive enhancement than for other purposes.

### Drug use motivations

Table 4 shows four of the most commonly indicated drug use motivations for modafinil, methylphenidate, Adderall and caffeine pills, by users of these substances (note that respondents could indicate more than one reason).

**Table 4:** Drug use purposes for modafinil, methylphenidate, Adderall and caffeine pills, as reported by users of these substances.

Substance Users	Enhance Cognition *n* (%)	Offset sleep deprivation *n* (%)	Enhance mood *n* (%)	Curiosity *n* (%)
modafinil, *n* = 70	54 (77.1%)	43 (61.4%)	12 (17.1%)	15 (21.4%)
methylphenidate, *n* = 52	35 (67.3%)	14 (26.9%)	10 (19.2%)	19 (36.5%)
Adderall, *n* = 28	18 (64.3%)	8 (28.6%)	6 (21.4%)	13 (46.4%)
caffeine pills, *n* = 432	213 (49.3%)	265 (61.3%)	46 (10.6%)	35 (8.1%)

Note that respondents could select more than one purpose for each drug.

All four substances were used, to varying degrees, to ‘enhance cognition’, ‘offset sleep deprivation’, and to ‘enhance mood’. Not included in Table 4 are the substances that were most commonly used to ‘enhance cognition’ and to ‘offset sleep deprivation’ by students in the sample as a whole (n = 877). These substances were energy drinks and caffeine tablets: 30% of the total sample had used energy drinks to enhance cognition; 45% had used energy drinks to offset sleep deprivation; 24% had used caffeine tablets to enhance cognition; 30% had used caffeine tablets to offset sleep deprivation.

### User groups characteristics

Considering methylphenidate, Adderall and modafinil together, two thirds of the sample was not interested in these drugs for any purpose. This category was broken down into three further groups. Fourteen percent of respondents said that they had never heard of any of the three smart drugs. Another 14.5% of students responded that they were never interested in any of the three drugs. The remaining 38% of respondents in this category were unaware of some, and uninterested in the other, substances.

Around 20% of students had considered using at least one of the three drugs, and 9.4% of the sample had used one of these drugs to improve cognitive performance at least once. About 3% of respondents had used one of these three drugs for purposes other than cognitive enhancement. Table 5 details this breakdown of the results.

**Table 5:** *n* = 877. User group categories on the basis of familiarity with methylphenidate, modafinil and Adderall.

User groups		*n* (%)
Unaware/not interested	Unaware of all PCEs	123 (14.0%)
	Uninterested in all PCEs	127 (14.5%)
	Unaware of some of the PCEs and uninterested in the other PCEs	336 (38.3%)
Considered using at least one PCE		179 (20.4%)
Non-CE type user (at least one drug)		30 (3.4%)
PCE-type user (at least one drug)		82 (9.4%)

### Self-reported ADHD-symptoms

In this sample, none of the four drug user groups received a score consistent with clinically significant symptomatology of adult ADHD (see Table 6).

**Table 6:** ADHD ASRS Score by user group, *n* = 877.

User groups		Mean	Std. Err.	95% confidence interval	
Unaware/not interested	Unaware of all PCEs	2.4	0.1	2.2	2.7
	Uninterested in all PCEs	2.4	0.1	2.1	2.7
	Unaware of some of the PCEs and uninterested in the other PCEs	2.6	0.1	2.4	2.7
Considered using at least one PCE		3.1	0.1	2.9	3.3
Non-CE type user (at least one drug)		3.1	0.3	2.4	3.7
PCE-type user (at least one drug)		2.7	0.2	2.3	3.1

### Conversion from interest in PCEs to use of PCEs

Table 7 details the three most common reported reasons given by respondents for why their interest in modafinil, methylphenidate and Adderall for PCE purposes did not convert to substance use. Lack of availability was the most common reason why interested students had not yet tried one of these three drugs, particularly in the case of the stimulant drugs. About half of students interested in PCE using stimulants cited lack of availability as the reason interest had not converted to use.

**Table 7:** Reasons for not using by those who have considered a PCE.

	Considered modafinil but not tried yet (*n* = 78)	Considered methylphenidate but not tried yet (*n* = 161)	Considered Adderall but not tried yet (*n* = 105)
	*n* (%)	*n* (%)	*n* (%)
**Lack of availability**	28 (35.9%)	83 (51.6%)	56 (53.3%)
**Concerns about side-effects**	13 (16.7%)	25 (15.5%)	14 (13.3%)
**Illegality**	9 (11.5%)	14 (8.7%)	11 (10.5 s%)

Note that only the three most common reasons are shown.

Not shown in the table is the finding that lack of availability was given as the sole reason why interest did not convert to use in a majority of cases (in 61/83 [74%] cases for methylphenidate, in 18/28 [64%] cases for modafinil, and in 40/56 [71%] cases for Adderall). The next most frequently cited reasons for non-conversion of interest to use were concerns about side effects, and concerns about the illegality of use.

Successful access to the stimulants (methylphenidate and Adderall), and modafinil was achieved via diverse routes. Adderall and methylphenidate were obtained primarily from friends. Modafinil was sourced primarily from an on-line distributor, such as an on-line pharmacy. Table 8 provides an overview of findings on the sources of PCEs.

**Table 8:** Source of PCEs reported by users of these substances.

Source of drug	Modafinil *n* (%)	Methylphenidate *n* (%)	Adderall *n* (%)
Family	0 (0%)	2 (4.1%)	1 (3.6%)
Friends	15 (21.4%)	37 (75.5%)	25 (89.3%)
Drug dealer	0 (0%)	2 (4.1%)	1 (3.6%)
Online	45 (64.3%)	1 (2.0%)	1 (3.6%)
Ambiguous entry	8 (11.4%)	7 (14.3%)	0 (0%)
**Total**	**69 (100%)**	**49 (100%)**	**28 (100%)**

### Ethics

The results of the broad ethics question are summarised in Table 9. Views on the ethics of PCE in academia were related to respondents' experience of and attitudes toward PCEs. Among those who were unaware of, or uninterested in, modafinil, Adderall and methylphenidate, 13% were neutral and a majority (69.2%) agreed or strongly agreed that PCE in academic contexts is ethically problematic. Respondents who had considered PCE, or who had used PCEs, found PCE in academia to be less problematic. Among respondents who had considered PCE, 20% were neutral about the ethics of PCE use in academia, and almost 45% agreed or strongly agreed that PCE use in academia was problematic. Among PCE users, about 16% were neutral and almost 21% agreed or strongly agreed that PCE use in academia is problematic. In comparison to other user groups, the aware but disinterested group tended to have stronger positive and stronger negative opinions about the ethics of PCE use in academic settings. A very small minority (6.4%) of respondents in this group strongly disagreed that PCE in academia is ethically problematic while just under half of respondents (45.7%) strongly agreed with the statement.

**Table 9:** PCE in academia is ethically problematic (*n* = 877).

	Unaware/uninterested in some PCEs *n* (%)	Completely unaware *n* (%)	Uninterested*n* (%)	Considered*n* (%)	Non-PCE-use *n* (%)	PCE-use *n* (%)
Strongly disagree	26 (7.8%)	16 (13.0%)	8 (6.4%)	32 (17.9%)	8 (26.7%)	32 (39.0%)
Disagree	34 (10.2%)	10 (8.1%)	5 (4.0%)	32 (17.9%)	6 (20%)	20 (24.4%)
Neutral	43 (12.8%)	16 (13.0%)	16 (12.6%)	35 (20.0%)	3 (10%)	13 (15.6.%)
Agree	108 (32.2%)	37 (30.1%)	40 (31.5%)	46 (25.7%)	7 (23.3%)	15 (18.3%)
Strongly agree	124 (37.0%)	44 (35.8%)	58 (45.7%)	34 (19.0%)	6 (20%)	2 (2.4%)
Total	335 (100%)	123 (100%)	127 (100%)	179 (100%)	30 (100%)	82 (100%)

Responses to the comparisons between PCE and doping in sports, and PCE and cheating were similarly dependent on user group, with those who had considered PCE and those who were PCE users less likely than the unaware/uninterested respondents to validate the comparisons. The comparison with cheating drew the strongest opinions from PCE users, 72% strongly disagreed with the comparison and no PCE users strongly agreed. Those who had considered PCE tended to disagree or strongly disagree that PCE was like cheating (29% and 32% respectively), or like doping in sports (21% and 22% respectively). About one fifth of those unaware/uninterested in PCE were neutral on both these comparisons. About one third of unaware/uninterested respondents disagreed to some extent with the comparison between PCE and cheating. However, 63% of these respondents endorsed the comparison between PCE and doping (27% agreed and 36% strongly agreed), and just under half of these respondents endorsed the comparison between PCE and cheating (24% agreed and 27% strongly agreed).

Across all user groups, a majority of respondents disagreed or strongly disagreed with the statement: “I would feel pressured to use PCEs if I knew other people used them.” The frequency of disagreement diminished as PCE became more of a reality, such that about 80% of the unaware/uninterested group disagreed or strongly disagreed with the statement; 53% of the group that had considered PCE use disagreed or strongly disagreed; and 52% of PCE users disagreed or strongly disagreed. Across groups, the highest proportion of respondents in agreement with the statement was found in the PCE user group; 22% of PCE users agreed with the statement; and 11% of PCE users agreed strongly with the statement. Those who were PCE users or who had considered PCE were more than twice as likely as other groups to agree that they might feel pressure to use PCEs.

### Predictors of PCE use

Tables 10–13 present the findings from a series of ordinal logistic regression models fitted for each of the modafinil, methylphenidate, Adderall and caffeine user groups.

**Table 10:** Ordinal logistic regression predicting knowledge and use of modafinil. +

Predictors	Model ∧∧					
	I	II	III	IV	V	VI
Female (reference category: male)	0.434***	0.435***	0.420***	0.414***	0.409***	0.450***
	(−6.20)	(−5.99)	(−6.17)	(−6.25)	(−6.26)	(−5.41)
age 21–24 (reference category: age 17–20)	2.001***	2.017***	1.951***	1.919***	1.911***	1.982***
	−4.56	−4.61	−4.35	−4.23	−4.20	−4.41
age 25–29 (reference category: age 17–20)	1.680*	1.682*	1.743*	1.718*	1.712*	1.670*
	−2.35	−2.34	−2.49	−2.42	−2.41	−2.29
age 30+ (reference category: age 17–20)	2.584**	2.491**	2.758***	2.748***	2.751***	2.703***
	−3.20	−3.05	−3.38	−3.36	−3.36	−3.30
Ethnicity: not-white (reference category: British white)	1.163	1.086	1.064	1.072	1.070	1.058
	−0.79	−0.41	−0.31	−0.35	−0.34	−0.28
Ethnicity: white but not British (reference category: British white)	0.597**	0.588**	0.566**	0.565**	0.566**	0.558**
	(−2.66)	(−2.73)	(−2.89)	(−2.90)	(−2.89)	(−2.96)
Alcohol use (ranges from 1/never tried to 4/use weekly or more)		0.912	0.930	0.935	0.937	0.938
		(−1.30)	(−1.01)	(−0.93)	(−0.90)	(−0.89)
Cannabis use (ranges from 1/never tried to 4/use weekly or more)		1.033	0.982	0.997	0.997	0.948
		−0.43	(−0.24)	(−0.04)	(−0.04)	(−0.68)
Awareness of other people at University using smart drugs (dichotomous: no and yes)			2.048***	2.049***	2.054***	1.997***
			−5.10	−5.10	−5.11	−4.89
ADHD score (ranges from 0 to 6)				0.939	0.930	0.927
				(−1.51)	(−1.62)	(−1.70)
Self-esteem score (ranges from 0 to 30)					0.993	0.993
					(−0.58)	(−0.54)
Beliefs about the ethnics of using smart drugs to improve academic performance (ranges from 1 to 5, where 1 means ‘strongly disagree’ and 5 means ‘strongly agree’ that it is ‘ethically problematic’)						0.867**
						(−2.62)
Sample size (*N*)	887	887	886	886	886	886

+ Outcome variable has four mutually exclusive categories: unaware, uninterested, have considered, and have used (including currently use) each drug.

∧∧ Parameter estimates are odd-ratios, i.e. exponentiated coefficients. Standard errors in parentheses.

*** p<.001,

** p<.01,

* p<.05.

**Table 11:** Ordinal logistic regression predicting knowledge and use of methylphenidate. +

Predictors	Model ∧∧					
	I	II	III	IV	V	VI
Female (reference category: male)	0.445***	0.568***	0.562***	0.579***	0.559***	0.635**
	(−5.99)	(−4.05)	(−4.10)	(−3.88)	(−4.09)	(−3.07)
age 21–24 (reference category: age 17–20)	1.513**	1.493**	1.410*	1.467*	1.451*	1.511**
	−2.80	−2.70	−2.29	−2.54	−2.47	−2.72
age 25–29 (reference category: age 17–20)	2.069***	1.920**	1.984**	2.030**	2.012**	1.990**
	−3.41	−3.02	−3.16	−3.27	−3.23	−3.18
age 30+ (reference category: age 17–20)	2.400**	2.542**	3.026***	3.033***	3.048***	2.960***
	−2.97	−3.13	−3.69	−3.71	−3.72	−3.65
Ethnicity: not-white (reference category: British white)	0.664*	0.841	0.817	0.812	0.810	0.802
	(−2.10)	(−0.85)	(−0.98)	(−1.01)	(−1.02)	(−1.07)
Ethnicity: white but not British (reference category: British white)	0.723	0.767	0.716	0.718	0.728	0.720
	(−1.76)	(−1.43)	(−1.79)	(−1.78)	(−1.70)	(−1.76)
Alcohol use (ranges from 1/never tried to 4/use weekly or more)		1.156*	1.202*	1.198*	1.205*	1.210**
		−2.01	−2.55	−2.49	−2.57	−2.61
Cannabis use (ranges from 1/never tried to 4/use weekly or more)		1.638***	1.545***	1.506***	1.511***	1.426***
		−6.60	−5.73	−5.36	−5.39	−4.51
Awareness of other people at University using smart drugs (dichotomous: no and yes)			2.485***	2.513***	2.533***	2.433***
			−6.46	−6.53	−6.57	−6.26
ADHD score (ranges from 0 to 6)				1.151***	1.121**	1.119*
				−3.39	−2.58	−2.53
Self-esteem score (ranges from 0 to 30)					0.978	0.979
					(−1.72)	(−1.68)
Beliefs about the ethics of using smart drugs to improve academic performance (ranges from 1 to 5, where 1 means ‘strongly disagree’ and 5 means ‘strongly agree’ that it is ‘ethically problematic’)						0.839**
						(−3.26)
Sample size (*N*)	887	887	886	886	886	886

+ Outcome variable has four mutually exclusive categories: unaware, uninterested, have considered, and have used (including currently use) each drug.

∧∧ Parameter estimates are odd-ratios, i.e. exponentiated coefficients. Standard errors in parentheses.

*** p<.001,

** * p*<.01,

* * p*<.05.

**Table 12:** Ordinal logistic regression predicting knowledge and use of Adderall. +

Predictors	Model ∧∧					
	I	II	III	IV	V	VI
Female (reference category: male)	0.520***	0.582***	0.581***	0.591***	0.568***	0.450***
	(−5.05)	(−4.04)	(−4.04)	(−3.90)	(−4.14)	(−5.41)
age 21–24 (reference category: age 17–20)	1.295	1.298	1.274	1.293	1.285	1.982***
	−1.80	−1.81	−1.68	−1.77	−1.73	−4.41
age 25–29 (reference category: age 17–20)	1.267	1.216	1.243	1.256	1.250	1.670*
	−1.15	−0.95	−1.05	−1.10	−1.08	−2.29
age 30+ (reference category: age 17–20)	0.740	0.715	0.771	0.771	0.778	2.703***
	(−0.98)	(−1.08)	(−0.83)	(−0.83)	(−0.80)	−3.300
Ethnicity: not-white (reference category: British white)	1.155	1.155	1.136	1.132	1.127	1.058
	−0.78	−0.75	−0.66	−0.64	−0.62	−0.28
Ethnicity: white but not British (reference category: British white)	1.185	1.192	1.157	1.164	1.174	0.558**
	−0.96	−0.99	−0.82	−0.85	−0.90	(−2.96)
Alcohol use (ranges from 1/never tried to 4/use weekly or more)		0.927	0.943	0.940	0.945	0.938
		(−1.10)	(−0.85)	(−0.88)	(−0.82)	(−0.89)
Cannabis use (ranges from 1/never tried to 4/use weekly or more)		1.283***	1.237**	1.223**	1.228**	0.948
		−3.47	−2.94	−2.76	−2.81	(−0.68)
Awareness of other people at University using smart drugs (dichotomous: no and yes)			1.627***	1.634***	1.645***	1.997***
			−3.68	−3.71	−3.75	−4.89
ADHD score (ranges from 0 to 6)				1.069	1.039	0.927
				−1.65	−0.88	(−1.70)
Self-esteem score (ranges from 0 to 30)					0.976	0.993
					(−1.94)	(−0.54)
Beliefs about the ethics of using smart drugs to improve academic performance (ranges from 1 to 5, where 1 means ‘strongly disagree’ and 5 means ‘strongly agree’ that it is ‘ethically problematic’)						0.867**
						(−2.62)
Sample size (*N*)	887	887	886	886	886	886

+ Outcome variable has four mutually exclusive categories: unaware, uninterested, have considered, and have used (including currently use) each drug.

∧∧ Parameter estimates are odd-ratios, i.e. exponentiated coefficients. Standard errors in parentheses.

*** p<.001,

** p<.01,

* p<.05.

**Table 13:** Ordinal logistic regression predicting knowledge and usage of caffeine. +

Predictors	Model ∧∧					
	I	II	III	IV	V	VI
Female (reference category: male)	0.731*	0.944	0.929	0.935	0.904	1.059
	(−2.41)	(−0.42)	(−0.53)	(−0.48)	(−0.72)	−0.39
age 21–24 (reference category: age 17–20)	1.131	1.109	1.069	1.075	1.063	1.110
	−0.86	−0.70	−0.45	−0.48	−0.41	−0.70
age 25–29 (reference category: age 17–20)	1.354	1.242	1.244	1.247	1.237	1.209
	−1.41	−0.99	−1.00	−1.01	−0.97	−0.85
age 30+ (reference category: age 17–20)	0.729	0.801	0.852	0.854	0.849	0.810
	(−1.05)	(−0.72)	(−0.52)	(−0.51)	(−0.53)	(−0.67)
Ethnicity: not-white (reference category: British white)	0.417***	0.549**	0.540**	0.539**	0.538**	0.519**
	(−4.67)	(−3.00)	(−3.08)	(−3.09)	(−3.10)	(−3.26)
Ethnicity: white but not British (reference category: British white)	0.514***	0.549***	0.536***	0.537***	0.540***	0.523***
	(−3.79)	(−3.34)	(−3.45)	(−3.44)	(−3.40)	(−3.56)
Alcohol use (ranges from 1/never tried to 4/use weekly or more)		1.265**	1.290***	1.288***	1.296***	1.302***
		−3.27	−3.51	−3.50	−3.57	−3.61
Cannabis use (ranges from 1/never tried to 4/use weekly or more)		1.659***	1.606***	1.595***	1.603***	1.492***
		−6.33	−5.87	−5.75	−5.79	−4.79
Awareness of other people at University using smart drugs (dichotomous: no and yes)			1.557**	1.556**	1.574***	1.492**
			−3.27	−3.26	−3.34	−2.92
ADHD score (ranges from 0 to 6)				1.030	1.003	1.000
				−0.71	−0.07	0.00
Self-esteem score (ranges from 0 to 30)					0.978	0.979
					(−1.70)	(−1.67)
Beliefs about the ethics of using smart drugs to improve academic performance (ranges from 1 to 5, where 1 means ‘strongly disagree’ and 5 means ‘strongly agree’ that it is ‘ethically problematic’)						0.809***
						(−3.86)
Sample size (*N*)	887	887	886	886	886	886

+ Outcome variable has four mutually exclusive categories: unaware, uninterested, have considered, and have used (including currently use) each drug.

∧∧ Parameter estimates are odd-ratios, i.e. exponentiated coefficients. Standard errors in parentheses.

*** *p*<.001,

** *p*<.01,

* *p*<.05.

Focusing on Model VI in each of the four tables (Tables 10–13), females and people in the youngest age group (ages 17–20) were less likely to use modafinil, methylphenidate and Adderall (but not caffeine) compared to males and the other age groups (conditioning on alcohol and cannabis use, awareness, beliefs about the ethics of using smart drugs to improve academic performance, and so on). White British students were more likely to use caffeine than non-White and non-British White students, and non-British White students were less likely than British White (and non-White) students to use modafinil. Alcohol and cannabis use were both positively associated with methylphenidate and with caffeine use, but this partial association did not hold for modafinil and Adderall.

ADHD symptoms were not generally related to smart drug use, although ADHD symptoms were a weak and positive predictor of methylphenidate use. Awareness of other people at university using smart drugs was strongly and positively associated with use of modafinil and methylphenidate and moderately associated with use of Adderall and caffeine.

Finally, we interpret the findings from multinomial logistic regressions for each of modafinil, methylphenidate and Adderall. The belief that it is unethical to use smart drugs to improve academic performance increased the predicted odds of moving from unaware to uninterested, but the same belief decreased the predicted odds of moving from uninterested to having considered taking the drug, and of moving from having considered taking the drug to actually having taken it.

## Discussion

This article has reported the findings from the smart drug survey (www.thesmartdrugstudy.com), which is the first comprehensive national survey on cognitive enhancement among students enrolled at UK and Irish universities. The survey investigated cognitive enhancement in four areas: prevalence, practices, motivations, and ethics.

We defined a ‘smart drug’ for participants in order to address the problem of ambiguity around the concept. In order to avoid conflation with prescription drug users, we excluded students with prescriptions for methylphenidate, modafinil and Adderall in the survey. We employed a dimensional approach to understanding PCE prevalence to distinguish the level of *current* and *ongoing* use of PCEs in the university setting from *past* and *occasional* use. A dimensional investigation of PCE use therefore minimizes the risk of over-estimating PCE as a current problem in university settings. Such an approach is also a better means of evaluating the extent to which PCE poses a risk of drug dependence, because it differentiates occasional drug practices from sustained PCE.

Despite anecdotal reports of high rates of PCE in UK universities, a majority of students surveyed in this study were unaware of and/or uninterested in PCEs. Collectively, students in these user groups made up 67% of the study sample.

PCE users were likely to be British male students nearing the end of an undergraduate degree course or at postgraduate level. Current users, and regular past users of methylphenidate and Adderall made up less than 1% of the study sample, respectively. Under 6% of students had used methylphenidate or Adderall as a cognitive enhancer at least once. Modafinil was the most commonly used PCE; just over 8% of the study sample had used modafinil at least once. Modafinil was the only drug for which the proportion of past users and current users was almost equal (4.1% and 3.9% of the study population, respectively). Methylphenidate, the most widely known PCE among surveyed students, was also the substance that students were least interested in trying as a cognitive enhancer (relative to modafinil, Adderall and caffeine).

While students have concerns about the ethics of PCE in the university, there is little indication of strong principled disagreement with PCE. In general, the level of concern about the ethics of PCE use varied with students' interest in and use of PCEs, with those who had considered PCE and used PCE reporting lower levels of ethical concern than those who were uninterested and/or unaware. Nevertheless, our findings suggest that moral considerations may be positively associated with a sustained lack of interest among students in accessing and in using PCEs.

Concerns about peer pressure or coercion to use PCEs were very low among all students. Interestingly, concern about coercion grew with interest in and with personal use of PCE; we also found that awareness of PCE use in the peer group strongly predicted personal use. Taken together, these findings suggest that direct and indirect peer pressure may be mechanisms by which PCE spreads within groups in the university context. Such group dynamics warrant further investigation, particularly as they may help to explain why PCE use in certain UK universities is anecdotally reported to be high, while PCE use across UK universities appears to be low [11].

In line with patterns of recreational drug use, curiosity motivated occasional PCE for those substances that were less available to students, perhaps because the stimulant drugs were more likely to be sourced opportunistically from friends and family. Alcohol and cannabis use were associated with methylphenidate use as a cognitive enhancer, giving some further weight to concerns about the intersection of PCE with recreational drug use. At the same time, methylphenidate PCE use was also weakly associated with ADHD symptomatology, suggesting that, in a subset of students, PCE may approach self-medication [31]. Cannabis is another substance associated with self-medication of ADHD [32]. The relationship between use of stimulants for PCE, ADHD symptomatology, and cannabis use is complex and requires focused investigation.

Our findings lend themselves to at least two plausible interpretations. One interpretation is that the data indicate substantial resilience to PCE among UK and Irish university students. Resilience here is defined as: low lifetime prevalence of PCE and very low levels of consistent use of PCEs, in a setting in which there is awareness of and interest in PCEs. Resilience has not been well documented or described in the literature on cognitive enhancement to date. On the basis of this study, resilience cannot be attributed wholly to a lack of interest in cognitive enhancement. Caffeine, delivered in tablets and energy drinks, was reported to be a common and consistently used cognitive enhancer. Further studies should differentiate attitudes to cognitive enhancement and attitudes toward *pharmacological* cognitive enhancement. Low uptake of PCE among UK and Irish university students may reflect concerns about drugs as the means of cognitive enhancement, rather than reflecting the desirability of cognitive enhancement as an end [33].

Another interpretation of the survey findings focuses on the relatively high proportion of students who showed an interest in PCE. Students who had considered using PCEs (20.4%) and students who had used a PCE at least once (9.4%) collectively made up one-third of the study sample. A key question here is why student interest in, and initial experimentation with PCE does not convert at higher rates to current ongoing use of PCEs. One clear answer from this survey is lack of availability of PCEs.

Upon closer examination, the data suggest a nuanced relationship between availability and PCE use. Availability is likely a key factor in different *patterns* of PCE use. These patterns of use are also moderated by awareness of PCEs. We note that despite the relatively greater availability of modafinil in the UK, it is still the most unknown PCE relative to methylphenidate and Adderall, with nearly 60% of students reporting that they had never heard of it. Although students reported more difficulty accessing the prescription stimulants, more students were past users of methylphenidate (44 students) than past users of modafinil (36 students). However, *current* modafinil users exceeded current methylphenidate users by a ratio of approximately 3∶1.

The ease of internet access to modafinil is probably a factor in the ongoing use of this drug, resulting in a relatively higher proportion of current modafinil users despite the substantial lack of awareness about and interest in the drug. Similarly, the lack of availability of methylphenidate and Adderall in the UK is a probable factor in the relatively lower rates of ongoing use of these substances for cognitive enhancement, despite higher levels of awareness of and interest in these drugs.

Finally, we note that these PCE use patterns might look very different if students found PCE to make a remarkable difference in their academic performance and achievement. We would expect to find a higher rate of consistent PCE use under such circumstances; more demand for, and wider availability of PCEs in universities; and a higher likelihood of conversion from interest to use. From the current use pattern data, we infer that students who use PCEs are not finding them to make a remarkable difference to their academic progress.

## Limitations

As noted above, this survey was not based on a random sample. Results of convenience sample surveys may be biased, due to participant self-selection and other factors. We used an on-line survey tool rather than traditional survey approaches, which were considered too costly and unfeasible due to access and administration barriers. On-line surveys can have advantages compared to paper-based surveys [34], including ease of access and guaranteed anonymity.

We were unable to conduct extensive reliability and validity tests of individual survey questions, beyond the efforts outlined in the methods section. Methodological inconsistency is a major barrier to comparisons of PCE studies across and within countries. We hope that transparent publication of our methods and findings on the survey website will enable further testing of the questions as well as more consistency among future surveys of PCE.

## Conclusion

In May, 2014 the BBC reported that the UK Advisory Council on the Misuse of Drugs would carry out a review of ‘smart drug use’ (www.news.live.**bbc**.co.uk/newsbeat/27207469). Our research represents the most comprehensive survey of PCE to date among UK and Irish university students. We found a mixed picture of high ‘resilience’ combined with relatively high interest in PCE. Lifetime prevalence of PCE using modafinil, methylphenidate or Adderall was 9.4%; past regular and current PCE users of these substances made up between 0.3%–4% of the survey population. PCE users were more likely to be male, older and British; and they were very likely to be aware of other PCE users. ADHD symptomatology was weakly correlated with use of methylphenidate for PCE. Principled ethical disagreement with PCE was low; those students who thought PCE was unethical were less likely to develop an interest in, or desire to access PCE. Use of stimulants and modafinil for non-PCE purposes was low; however, alcohol and cannabis use predicted PCE.

The present study suggests two simultaneous paths for universities to take in relation to PCE: monitoring and education. UK and Irish universities should discuss reasons and strategies to monitor PCE availability and circulation. On the basis of this study, we feel it would be unreasonable for universities to institute drastic monitoring measures, because students show themselves to be sufficiently resilient to PCE without direct intervention. Moreover, university student life is ideally characterised by increasing autonomy and responsibility; these values should not be undermined through PCE surveillance activities.

Universities should take account of the level of interest in cognitive enhancement and educate students to make responsible decisions about PCEs. We recognise that ‘responsible’ decision-making in relation to misuse of prescription drugs, could be taken to mean that in all cases students should not misuse such drugs for PCE. However, we favour a pragmatic approach in which ‘responsible decision-making’ is characterised by raising awareness of the ethics, risks and benefits of pharmacological means of cognitive enhancement, relative to other means. As part of this endeavour, universities should focus on deflating the media hype around PCE and correcting the wrong impression that sustained use of ‘smart drugs’ is highly prevalent among UK students.
